# Carbon dioxide (CO_2_) emissions and adherence to Mediterranean diet in an adult population: the Mediterranean diet index as a pollution level index

**DOI:** 10.1186/s12940-022-00956-7

**Published:** 2023-01-05

**Authors:** Silvia García, Cristina Bouzas, David Mateos, Rosario Pastor, Laura Álvarez, María Rubín, Miguel Ángel Martínez-González, Jordi Salas-Salvadó, Dolores Corella, Albert Goday, J. Alfredo Martínez, Ángel M. Alonso-Gómez, Julia Wärnberg, Jesús Vioque, Dora Romaguera, José Lopez-Miranda, Ramon Estruch, Francisco J. Tinahones, José Lapetra, Lluís Serra-Majem, Blanca Riquelme-Gallego, Xavier Pintó, José J. Gaforio, Pilar Matía, Josep Vidal, Clotilde Vázquez, Lidia Daimiel, Emilio Ros, Maira Bes-Rastrollo, Patricia Guillem-Saiz, Stephanie Nishi, Robert Cabanes, Itziar Abete, Leire Goicolea-Güemez, Enrique Gómez-Gracia, Antonio José Signes-Pastor, Antoni Colom, Antonio García-Ríos, Sara Castro-Barquero, Jose C. Fernández-García, José Manuel Santos-Lozano, Zenaida Vázquez, José V. Sorlí, Maria Pascual, Olga Castañer, Maria Angeles Zulet, Jessica Vaquero-Luna, F. Javier Basterra-Gortari, Nancy Babio, Ramon Ciurana, Vicente Martín-Sánchez, Josep A. Tur

**Affiliations:** 1 CIBER Fisiopatología de la Obesidad y Nutrición (CIBEROBN), Instituto de Salud Carlos III (ISCIII), 28029 Madrid, Spain; 2 Research Group on Community Nutrition & Oxidative Stress, University of Balearic Islands-IUNICS, Guillem Colom Bldg, Campus, E-07122 Palma de Mallorca, Spain; 3 Health Research Institute of the Balearic Islands (IdISBa), 07120 Palma de Mallorca, Spain; 4 Faculty of Health Sciences, Catholic University of Avila, 05005 Avila, Spain; 5 CIBER Epidemiología y Salud Pública (CIBERESP), Instituto de Salud Carlos III (ISCIII), 28029 Madrid, Spain; 6 Institute of Biomedicine (IBIOMED), University of León, 24071 Leon, Spain; 7 Department of Preventive Medicine and Public Health, IDISNA, University of Navarra, 31008 Pamplona, Spain; 8 Department of Nutrition, Harvard T. H. Chan School of Public Health, Boston, USA; 9 Biochemistry and Biotechnology Department, Human Nutrition Unit, IISPV, Universitat Rovira i Virgili, Hospital Universitari de Sant Joan, 43201 Reus, Spain; 10 Department of Preventive Medicine, University of Valencia, 46100 Valencia, Spain; 11 Unit of Cardiovascular Risk and Nutrition, Institut Hospital del Mar de Investigaciones Médicas Municipal d’Investigació Mèdica (IMIM), 08003 Barcelona, Spain; 12 Department of Medicine, University Autonomous of Barcelona, 08003 Barcelona, Spain; 13 Cardiometabolics Precision Nutrition Program, IMDEA Food, CEI UAM + CSIC, 28049 Madrid, Spain; 14 Department of Nutrition, Food Sciences, and Physiology, Center for Nutrition Research, University of Navarra, 31008 Pamplona, Spain; 15 Bioaraba Health Research Institute; Osakidetza Basque Health Service, Araba University Hospital; University of the Basque Country UPV/EHU, 48013 Vitoria-Gasteiz, Spain; 16 Department of Nursing, School of Health Sciences, University of Málaga-IBIMA, 29071 Málaga, Spain; 17 Instituto de Investigación Sanitaria y Biomédica de Alicante, Universidad Miguel Hernández (ISABIAL-UMH), 03550 Alicante, Spain; 18 Lipids and Atherosclerosis Unit, Department of Internal Medicine, Maimonides Biomedical Research Institute of Cordoba (IMIBIC), Reina Sofia University Hospital, University of Cordoba, 14004 Córdoba, Spain; 19 Department of Internal Medicine, IDIBAPS, Hospital Clinic, University of Barcelona, 08036 Barcelona, Spain; 20 Virgen de la Victoria Hospital, Department of Endocrinology, University of Málaga, 29010 Málaga, Spain; 21 Department of Family Medicine, Research Unit, Distrito Sanitario Atención Primaria Sevilla, 41013 Sevilla, Spain; 22 Institute for Biomedical Research, University of Las Palmas de Gran Canaria, 35016 Las Palmas, Spain; 23 Department of Preventive Medicine, University of Granada, 18071 Granada, Spain; 24 Lipids and Vascular Risk Unit, Internal Medicine, Hospital Universitario de Bellvitge, Hospitalet de Llobregat, 08907 Barcelona, Spain; 25 Department of Health Sciences, Center for Advanced Studies in Olive Grove and Olive Oils, University of Jaen, 23071 Jaen, Spain; 26 Department of Endocrinology and Nutrition, Instituto de Investigación Sanitaria San Carlos (IdISSC), 28040 Madrid, Spain; 27 Department of Endocrinology, IDIBAPS, Hospital Clinic, University of Barcelona, 08036 Barcelona, Spain; 28 Department of Endocrinology, Fundación Jiménez-Díaz, 28040 Madrid, Spain; 29 Nutritional Control of the Epigenome Group, Precision Nutrition and Obesity Program.IMDEA Food, CEI UAM + CSIC, 28049 Madrid, Spain; 30 Lipid Clinic, Department of Endocrinology and Nutrition, Institut d’Investigacions Biomèdiques August Pi Sunyer (IDIBAPS), Hospital Clínic, 08036 Barcelona, Spain; 31 Toronto 3D (Diet, Digestive Tract and Disease) Knowledge Synthesis and Clinical Trials Unit, Toronto, ON Canada; 32 Clinical Nutrition and Risk Factor Modification Centre, St. Michael’s Hospital, Unity Health Toronto, Toronto, ON Canada; 33 Department of Public Health and Psychiatry, School of Medicine, University of Malaga, and Biomedical Research Institute of Malaga (IBIMA), 29010 Málaga, Spain; 34 Department of Endocrinology, Hospital Universitario de Navarra, Osasunbidea, 31008 Pamplona, Spain

**Keywords:** Greenhouse gas emissions, Mediterranean diet, Carbon dioxide, Sustainability, Sustainable diets, Environment

## Abstract

**Background:**

Research related to sustainable diets is is highly relevant to provide better understanding of the impact of dietary intake on the health and the environment.

**Aim:**

To assess the association between the adherence to an energy-restricted Mediterranean diet and the amount of CO_2_ emitted in an older adult population.

**Design and population:**

Using a cross-sectional design, the association between the adherence to an energy-reduced Mediterranean Diet (erMedDiet) score and dietary CO_2_ emissions in 6646 participants was assessed.

**Methods:**

Food intake and adherence to the erMedDiet was assessed using validated food frequency questionnaire and 17-item Mediterranean questionnaire. Sociodemographic characteristics were documented. Environmental impact was calculated through greenhouse gas emissions estimations, specifically CO_2_ emissions of each participant diet per day, using a European database. Participants were distributed in quartiles according to their estimated CO_2_ emissions expressed in kg/day: Q1 (≤2.01 kg CO_2_), Q2 (2.02-2.34 kg CO_2_), Q3 (2.35-2.79 kg CO_2_) and Q4 (≥2.80 kg CO_2_).

**Results:**

More men than women induced higher dietary levels of CO_2_ emissions. Participants reporting higher consumption of vegetables, fruits, legumes, nuts, whole cereals, preferring white meat, and having less consumption of red meat were mostly emitting less kg of CO_2_ through diet. Participants with higher adherence to the Mediterranean Diet showed lower odds for dietary CO_2_ emissions: Q2 (OR 0.87; 95%CI: 0.76-1.00), Q3 (OR 0.69; 95%CI: 0.69-0.79) and Q4 (OR 0.48; 95%CI: 0.42-0.55) vs Q1 (reference).

**Conclusions:**

The Mediterranean diet can be environmentally protective since the higher the adherence to the Mediterranean diet, the lower total dietary CO_2_ emissions. Mediterranean Diet index may be used as a pollution level index.

## Introduction

Despite law regulations issued in the last few decades, greenhouse gas (GHG) emissions have increased, affecting climate change as well as the way of life. Carbon dioxide (CO_2_) represents one of the main GHG. As such, its reduction is part of the United Nations agenda 2030 which, in general terms, aims to eradicate poverty and promote sustainable and equalitarian development by 2030 following 17 sustainable development goals [1].

Global dietary patterns have changed too, and a new lifestyle characterized as quick and stressful has affected our way of purchasing and eating food, causing a detrimental impact on our health. This new way of living has also changed due to the increasing demand of meat protein, driven by the increasing of annual incomes in the last decades. A demand of empty calories found in products like refined cereals, refined sugars, alcohol, and oils was another of the global changes. Finally, the total per capita caloric demand increased as well [2].

New food habits and dietary changes have affected the amount of CO_2_ in the atmosphere, since food system emissions are around 1/3 of the global GHG emissions, representing 34% of total CO_2_ equivalents in 2015 [3]. Each increased step in the food chain has an added impact on the degradation of the environment. The production step has a particular impact, and this is the reason why the Eat Lancet Commission established that major changes must be made on both the way we eat and the way we produce our food to stop this detrimental situation [4].

Accordingly, there is a diet-environmental-health trilemma and research on how to be more sustainable and reduce those impacts has been increasing. Sustainable diets are those with low environmental impacts which contribute to food and nutrition security and to a healthy life for present and future generations. These types of diets are protective and respectful of biodiversity and ecosystems, culturally acceptable, accessible, economically fair, and affordable; nutritionally adequate, safe and healthy, while optimizing natural and human resources [5].

The traditional Mediterranean diet is a well-studied model in terms of healthfulness being researched for its protective effects on cardiometabolic risk factors and reducing the incidence on major cardiovascular events in a high-risk population [6]. Nowadays, cardiovascular diseases are the main cause of death in developed countries as well as in Spain [7]. According to the World Heart Federation, tobacco, hypertension, hypercholesterolemia, obesity, diabetes, physical inactivity, and inadequate diet are the main cardiovascular disease risk factors [8]. Due to its beneficial effects on cardiovascular health, Mediterranean diet is commonly recommended.

The Mediterranean Diet is characterized by a high consumption of fruits and vegetables, unrefined cereals, plant-origin proteins, and healthy fats such as olive oil, nuts, and fatty fish. A low consumption of animal products, mainly red and processed meat, which is one of the main contributors to CO_2_ emissions, is also one of the key traits of the characteristic points of the Mediterranean diet. Limiting overconsumption and energy intakes to an amount that meets recommendations was proposed as another possible beneficial aspect for reducing the impact on the ecosystems, and this also may help to decrease the obesity epidemic [9].

People following the Mediterranean Diet are already beneficiated for its protective effects on health. It would be interesting to study if people following the Mediterranean Diet are also protecting the environment while reducing CO_2_ emissions. The present study offers an opportunity to assess the association between the adherence to an energy-restricted Mediterranean diet and the amount of CO_2_ emitted in an older adult population.

## Methodology

### Study design

The present research was a cross-sectional analysis of baseline data within an ongoing 8-year multicenter, parallel-group, randomized trial, conducted in 23 Spanish recruiting centers aiming to assess the effect of weight-loss induced by a hypocaloric traditional Mediterranean Diet combined with physical activity promotion and behavioral support on cardiovascular disease and mortality. The study protocol can be found elsewhere [10]. The trial was registered in 2014 at the International Standard Randomized Controlled Trial (ISRCT; http://www.isrctn.com/ISRCTN89898870) with number 89898870.

### Participants, recruitment, and ethics

**Fig. 1:**
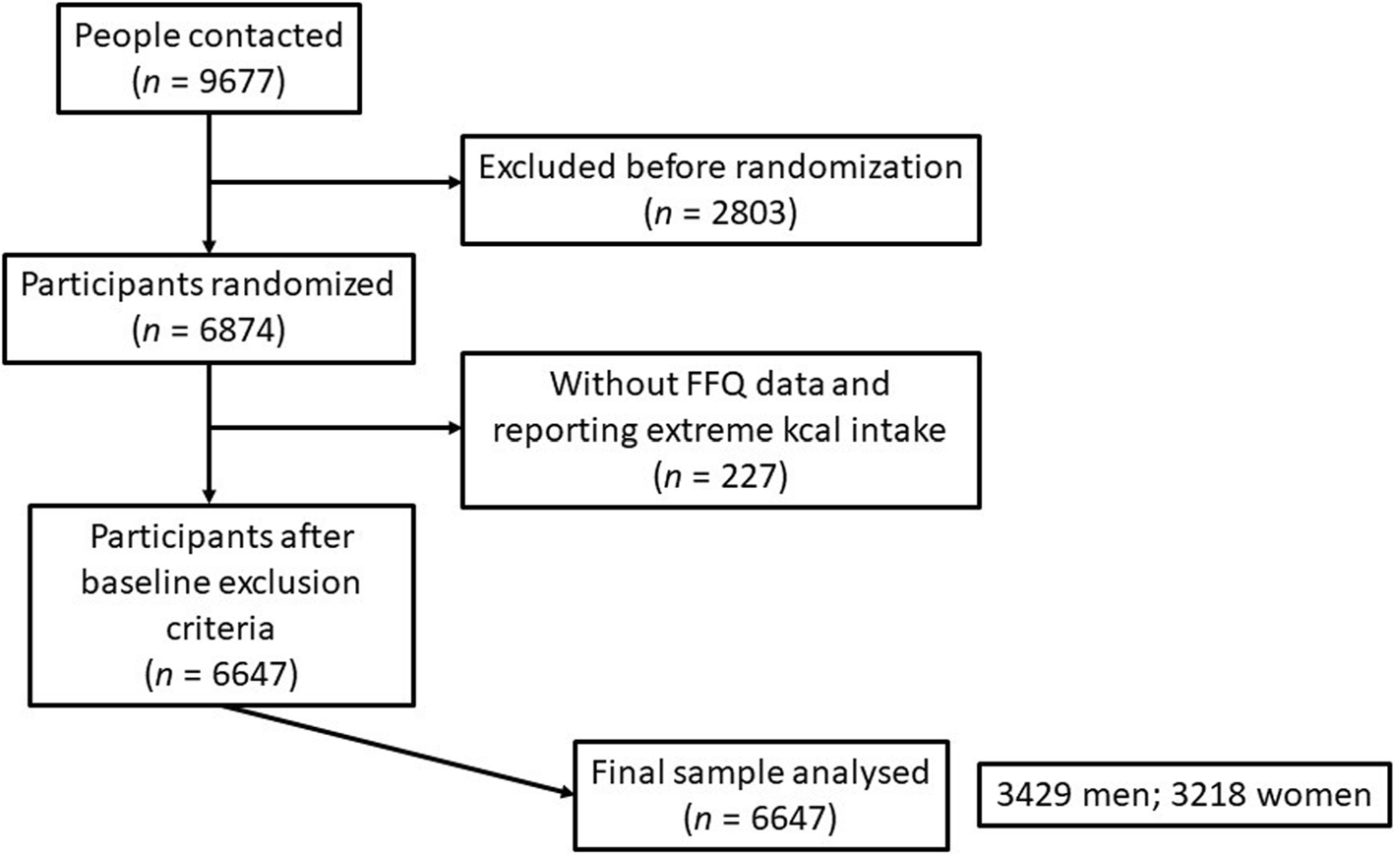
Flow chart of eligibility of participants

A total of 9677 participants were contacted; 6874 participants met the inclusion criteria including men aged 55-76 and women aged 60-75, with overweight or obese (body mass index between 27 and 40 kg/m^2^) and meeting at least three criteria for metabolic syndrome according to the Association and National Heart, Lung and Blood Institute [11]. Finally, 6646 participants were included in the analysis after excluding those with incomplete FFQ data and reporting extreme energy intakes (< 500 or > 3500 kcal/day in women or < 800 or > 4000 kcal/day in men) [12]. A flow-chart of eligible participants was shown in Fig. 1.

Informed written consent was provided by all participants and the study protocol and procedures were approved by ethical committees according to the ethical standards of the Declaration of Helsinki by all the 23 participating institutions.

### Assessment of dietary intake

Registered dietitians assessed dietary habits, at baseline, through a semi quantitative 143-item food frequency questionnaire (FFQ) [13] which has been previously validated in Spanish population [13–15]. For each item, a regular portion size was established, and consumption frequencies were registered according to 9 categories, ranging from “never or almost never” to “≥6 times/day”. Energy and nutrient intakes were calculated as frequency multiplied by nutrient composition of specified portion size for each food item, using a computer program based on available information from Spanish food composition tables [16, 17]. The results were used to determine the specific amount of food (in grams) each participant had eaten per day.

### CO_2_ emitted per kg of food

The amount of CO_2_ emitted per kg of consumed food per participant and day was calculated using a European database from 2016 that described kg of CO_2_ emitted per kg of food consumed. This database was based on life cycle assessment (LCA) of recent studies and included agricultural production and processing steps (considering defaults for cooking, storing, and packing and letting transportation out of the calculations) [18]. Kilograms of CO_2_ emitted per consumed food were calculated by multiplying g of each consumed food reported from the FFQ per kg of CO_2_ emitted per kg of each food from the database. The sum of all kilograms of CO_2_ emitted for all the products was done to determine the total emissions a day from diet. Once the CO_2_ emitted for each participant was known, an adjustment per 1 kg of food consumed was completed. The adjustment was done to consider the energy intake cofounder. Depending on the individual needs, the dietary intake could be higher in terms of quantity meaning higher emissions, even when comparing diets based on the same products. Therefore, an adjustment per 1 kg of food product per person offers a better comparison between the emissions of the participants’ diets and avoids bias for people who could eat higher amounts due to their personal needs.

### Assessment of adherence to the erMedDiet

Adherence to energy-reduced Mediterranean diet was assessed using a 17-item Mediterranean Diet validated questionnaire [19].

### Other health variables

Information related to sociodemographic characteristics such as sex, age, and scholar level were self-reported. Anthropometric measurements (including weight, height, waist, and hip circumference) were obtained.

### Statistical analyses

Analyses were performed using SPSS statistical software package version 27.0 (SPPS Inc., Chicago, IL, USA). Data are shown as mean and standard deviation (SD), except for prevalence data, which was expressed as sample size and percentage. Chi-squared test was used for categorical variables and one-way ANOVA and Bonferroni’s post-hoc was used for continuous variables. To assess the linear trend, the median value of each quartile of CO_2_ emissions was assigned and used as a continuous variable in the logistic regression model. Logistic regression was fitted to assess association between each one of the 17-items of erMedDiet questionnaire and the mean adherence to the Mediterranean Diet (as dependent variables) and quartiles of dietary CO_2_ emitted (as independent variable) calculating Odds Ratio (OR) value, crude and adjusted (by sex, age, and educational level). Data on the amount of CO_2_ emissions per participant and day were distributed in quartiles: quartile 1 (Q1); participants with the lowest emissions (≤2.01 kg CO_2_/day), quartile 2 (Q2); participants with low-moderate emissions (2.02-2.34 kg CO_2_/day), quartile 3 (Q3); participants with moderate-high emissions (2.35-2.79 kg CO_2_/day) and quartile 4 (Q4); participants with the highest emissions (≥2.80 kg CO_2_/day). Q1 was considered as the reference. A linear prediction with a 95% confidence interval (CI) was calculated between quartiles of dietary CO_2_ and the erMedDiet adherence score.

## Results

Table 1 shows the association between sex, age, scholar level, and adherence to the erMedDiet according to the kg CO_2_ emissions per kg of food. More men than women were classified into quartiles 3 (Q3) and 4 (Q4), which shows higher levels of CO_2_ emissions in men’s diets. Compared to those in the lowest quartile of kg CO_2_ emissions per kg of food, participants in the top quartile were more likely to be men, younger and with lower education level.

**Table 1:** Sex, scholar level, age, and adherence to the Mediterranean Diet according to CO_2_ emissions (quartiles)

	Q1 § *n* = 1661	Q2 § *n* = 1662	Q3 § *n* = 1663	Q4 § *n* = 1660	*p*-value
**Sex**					
Men	788 (47.4)	824 (49.6)	884 (53.2)	932 (56.1)	< 0.001
Women	873 (52.6)	838 (50.4)	779 (46.8)	728 (43.9)	
**Highest scholar level**					
Bachelor’s degree	201 (12.1)	202 (12.2)	214 (12.9)	241 (14.5)	< 0.001
College School Technician	143 (8.6)	164 (9.9)	138 (8.3)	155 (9.3)	
Secondary School	415 (25)	456 (27.4)	499 (30)	548 (33)	
Primary School	902 (54.3)	840 (50.5)	812 (48.8)	716 (43.1)	
**Age (years)**	65.2 (4.9)^c^	65.1 (4.9)	64.8 (4.9)	64.8 (4.9)^c^	0.02
**Weight (kg)**	85.4 (12.8)^b c^	86.1 (12.8)	86.8 (12.8)^b^	87.8 (13.4)^c^	< 0.001
**BMI (kg/m**^**2**^**)**	32.5 (3.5)^c^	32.5 (3.4)	32.5 (3.5)	32.7 (3.5)^c^	0.039
**Energy intake (Kcal/day)**	2329 (582.2)^c^	2358.4 (527.8)	2368.6 (538.1)	2404.8 (554)^c^	0.001
**MedDiet Adherence**					
Low adherence (0-8)	715 (43)	771 (46.4)	869 (52.3)	1013 (61)	< 0.001
High adherence (9-17)	946 (57)	891 (53.6)	794 (47.7)	647 (39)	

Data are expressed in n (%) or mean (standard deviation)

*Abbreviations*: *BMI* Body Mass Index, *MedDiet* Mediterranean Diet

§Kg of CO_2_ per consumed food = Kg of each consumed food from FFQ*Kg CO_2_ emitted per kg of each food (EU data base 2016). Four groups were considered according to CO_2_ emissions: Q1: ≤2.01 kg CO_2_/day; Q2: 2.02-2.34 kg CO_2_/day; Q3: 2.35-2.80 kg CO_2_/day; Q4: > 2.80 kg CO_2_/day. Differences between groups were assessed by chi-square for categorical variables and difference in means between groups were tested by one-way ANOVA and Bonferroni’s post-hoc for age

Bonferroni's post-hoc differences: b: Q1 vs Q3; c: Q1 vs Q4

The association between the adherence to the erMedDiet and its components across of quartiles of kg CO_2_ emissions per kg of food are showed in Table 1. Adherence to the erMedDiet was inversely associated across quartile of kg CO_2_ emissions per kg of food. A higher number of participants reporting higher adherence to the Mediterranean Diet were found in Q1 and Q2.

Crude and adjusted OR for adherence to Mediterranean Diet is shown in Table 2. Q1 (≤2.01 kg CO_2_) was the reference, and the adjustment was done by sociodemographic characteristics (sex, age, and scholar level). Crude and adjusted OR values on total Mediterranean Diet adherence were lower in both Q3 (OR 0.69 0.60-0.79) and Q4 (0.48 0.42-0.55) than in Q2 (OR 0.87 0.76-1.00), which means that participants high followers of Mediterranean Diet showed lower amount of CO2 emissions.

**Table 2:** Association (Odd Ratio and 95% Confidence interval) between the adherence to the energy restricted Mediterranean diet components (dependent variables) and quartiles of dietary kg CO_2_ emissions (independent variables)

		Q1 § ≤2.01 kg CO_2_/day *n* = 1661	Q2 § 2.02-2.34 kg CO_2_/day *n* = 1662	Q3 § 2.35-2.80 kg CO_2_/day *n* = 1663	Q4 § > 2.80 kg CO_2_/day *n* = 1660	*p* for trend
**erMedDiet 17-items**						
1.EVOO for cooking	*Crude OR*	1.00 (Ref.)	1.00 (0.84-1.18)	0.95 (0.80-1.13)	0.94 (0.79-1.11)	0.821
	*Adjusted OR*	1.00 (Ref.)	0.98 (0.83-1.16)	0.93(0.78-1.10)	0.89 (0.75-1.05)	0.507
2.Vegetables	*Crude OR*	1.00 (Ref.)	0.98 (0.86-1.13)	0.84 (0.73-0.96)	0.75 (0.65-0.87)	< 0.001
	*Adjusted OR*	1.00 (Ref.)	0.99 (0.86-1.14)	0.85 (0.74-0.98)	0.77 (0.67-0.89)	0.001
3.Fruits	*Crude OR*	1.00 (Ref.)	0.81 (0.71-0.93)	0.61 (0.54-0.71)	0.47 (0.41-0.55)	< 0.001
	*Adjusted OR*	1.00 (Ref.)	0.82 (0.72-0.94)	0.64 (0.55-0.73)	0.50 (0.43-0.57)	< 0.001
4.Red and processed meat	*Crude OR*	1.00 (Ref.)	0.65 (0.56-0.74)	0.46 (0.40-0.52)	0.29 (0.25-0.33)	< 0.001
	*Adjusted OR*	1.00 (Ref.)	0.65 (0.57-0.75)	0.47 (0.41-0.54)	0.30 (0.26-0.35)	< 0.001
5.Butter, margarine, cream	*Crude OR*	1.00 (Ref.)	1.18 (0.99-1.40)	1.05 (0.89-1.25)	1.11 (0.94-1.31)	0.275
	*Adjusted OR*	1.00 (Ref.)	1.18 (0.99-1.40)	1.05 (0.89-1.24)	1.10 (0.92-1.30)	0.305
6.Sugar sweetened beverages	*Crude OR*	1.00 (Ref.)	1.05 (0.89-1.23)	0.95 (0.81-1.11)	0.97 (0.83-1.13)	0.610
	*Adjusted OR*	1.00 (Ref.)	1.06 (0.90-1.24)	0.97 (0.83-1.13)	1.00 (0.85-1.17)	0.758
7.Legumes	*Crude OR*	1.00 (Ref.)	0.75 (0.67-0.89)	0.62 (0.52-0.73)	0.65 (0.54-0.77)	< 0.001
	*Adjusted OR*	1.00 (Ref.)	0.75 (0.64-0.89)	0.62 (0.52-0.73)	0.65 (0.55-0.77)	< 0.001
8.Fish and seafood	*Crude OR*	1.00 (Ref.)	1.41 (1.23-1.61)	1.39 (1.21-1.59)	1.46 (1.27-1.67)	< 0.001
	*Adjusted OR*	1.00 (Ref.)	1.41 (1.23-1.62)	1.41 (1.23-1.62)	1.48 (1.29-1.70)	< 0.001
9.Sweets and pastries	*Crude OR*	1.00 (Ref.)	0.92 (0.80-1.06)	0.92 (0.80-1.06)	0.92 (0.80-1.05)	0.538
	*Adjusted OR*	1.00 (Ref.)	0.92 (0.80-1.06)	0.92 (0.80-1.06)	0.92 (0.80-1.06)	0.557
10.Nuts	*Crude OR*	1.00 (Ref.)	0.97 (0.85-1.11)	0.93 (0.81-1.07)	0.77 (0.67-0.88)	0.001
	*Adjusted OR*	1.00 (Ref.)	0.96 (0.84-1.10)	0.92 (0.80-1.06)	0.75 (0.65-0.86)	< 0.001
11.Preference white over red meat	*Crude OR*	1.00 (Ref.)	0.85 (0.71-1.01)	0.56 (0.47-0.66)	0.27 (0.23-0.31)	< 0.001
	*Adjusted OR*	1.00 (Ref.)	0.86 (0.72-1.03)	0.58 (0.49-0.69)	0.28 (0.24-0.33)	< 0.001
12.Sofrito	*Crude OR*	1.00 (Ref.)	0.96 (0.83-1.10)	0.93 (0.81-1.07)	0.68 (0.59-0.77)	< 0.001
	*Adjusted OR*	1.00 (Ref.)	0.96 (0.83-1.10)	0.93 (0.81-1.07)	0.68 (0.59-0.78)	< 0.001
13.Adding sugar to beverages	*Crude OR*	1.00 (Ref.)	1.02 (0.89-1.18)	0.91 (0.79-1.05)	1.07 (0.93-1.23)	0.171
	*Adjusted OR*	1.00 (Ref.)	1.03 (0.89-1.19)	0.94 (0.81-1.08)	1.10 (0.96-1.28)	0.161
14.White bread	*Crude OR*	1.00 (Ref.)	0.92 (0.80-1.05)	0.85 (0.74-0.98)	0.87 (0.76-1.00)	0.099
	*Adjusted OR*	1.00 (Ref.)	0.92 (0.80-1.06)	0.87 (0.76-1.00)	0.90 (0.78-1.03)	0.243
15.Whole grains	*Crude OR*	1.00 (Ref.)	0.91 (0.79-1.06)	0.74 (0.64-0.86)	0.54 (0.46-0.64)	< 0.001
	*Adjusted OR*	1.00 (Ref.)	0.91 (0.79-1.06)	0.75 (0.65-0.88)	0.55 (0.47-0.64)	< 0.001
16.Refined cereals	*Crude OR*	1.00 (Ref.)	0.93 (0.80-1.07)	0.90 (0.78-1.04)	0.80 (0.69-0.93)	0.029
	*Adjusted OR*	1.00 (Ref.)	0.93 (0.80-1.08)	0.92 (0.80-1.07)	0.82 (0.71-0.95)	0.080
17.Wine	*Crude OR*	1.00 (Ref.)	1.48 (1.25-1.75)	1.51 (1.27-1.78)	1.34 (1.14-1.59)	< 0.001
	*Adjusted OR*	1.00 (Ref.)	1.47 (1.24-1.75)	1.44 (1.21-1.71)	1.23 (1.03-1.46)	< 0.001
**MedDiet Adherence**	*Crude OR*	1.00 (Ref.)	0.87 (0.76-1.00)	0.69 (0.60-0.79)	0.48 (0.42-0.55)	< 0.001
	*AdjustedOR*	1.00 (Ref.)	0.87 (0.76-1.00)	0.71 (0.61-0.81)	0.49 (0.43-0.56)	< 0.001

*Abbreviations*: *erMedDiet* energy-reduced Mediterranean diet, *EVOO* Extra Virgin Olive Oil, *MedDiet* Mediterranean Diet, *OR* Odds Ratio, *Adjusted OR* Odds Ratio adjusted by sociodemographic characteristics (sex, scholar level and age).

§Kg of CO_2_ per consumed food = Kg of each consumed food from FFQ*Kg CO_2_ emitted per kg of each food (EU data base 2016)

**Fig. 2:**
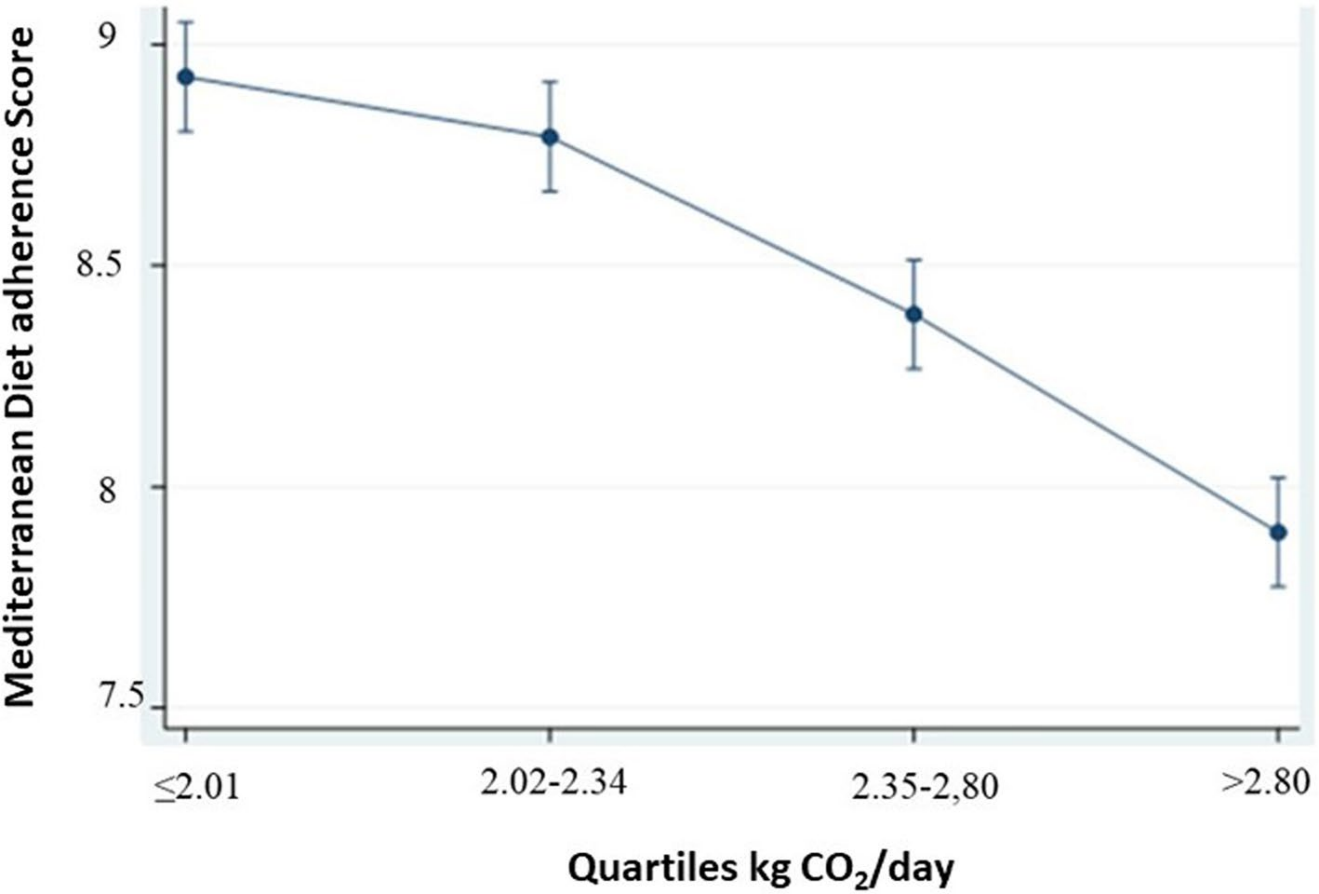
Predictive margins of kg CO_2_ quartiles and the Mediterranean Diet adherence score with 95%CI

Figure 2 shows that there was lower adherence to the Mediterranean diet in those participants with higher CO_2_ emissions.

## Discussion

The current study showed that CO_2_ emissions were inversely associated with the adherence to the Mediterranean Diet. It also opened the idea of using the erMedDiet index as a pollution level index. Studies in younger populations have already shown how a Mediterranean Diet could be proposed as a sustainable dietary model in terms of food production and processing. Better adherence to the Mediterranean Diet has been associated with lower land use, water consumption, energy consumption and GHG emissions [20]. Another study in Italian children compared the CO_2_ emissions of the Mediterranean Diet between winter and spring; impacts were higher in winter than in spring, and meat products were the major contributors to GHG emissions in both seasons, followed by milk and dairy products [21].

The scope of several studies has compared the Mediterranean Diet with other dietary patterns. A Western Diet (WD), characterized by a high consumption of meat, sweets, and beverages, appears to be the unhealthiest and the most detrimental pattern in terms of the environment, but the most affordable [22]. Compared to a Western Dietary pattern, the Mediterranean Diet in Spain would substantially reduce GHG emissions, land use and energy consumption, and lower extent water consumption [23]. Moreover, GHG emissions were lower for Mediterranean Diet pattern with a consistent emission 14.55% below to an Italian average diet and 6.74% below the Mediterranean Diet [24]. Compared to the DASH or Nordic Diets, higher adherence to the Mediterranean Diet has been associated with lower GHG emissions [25].

Other studies have calculated the environmental impact of different dietary scenarios, mainly based on healthy recommendations or food based dietary guidelines [26–28] or trying to represent a specific diet of a country [29, 30]. A reduction in premature mortality and a reduction in GHG emissions were seen in the healthy and sustainable diets [26, 29]. A common factor of those dietary scenarios was the reduction in animal-based products with an increase focus on plant-based foods [25–29]. This is relevant to the present analyses, as the Mediterranean Diet is a plant-forward dietary pattern because it emphasizes consumption of fruits and vegetables, healthy fats, whole cereals, as well as a preference for fish and white meat, with an overall reduction in red and processed meat [31].

Several studies have evaluated the environmental impact of a diet related to a specific country or a region, for instance, Switzerland [32], China [33–35], France, United Kingdom, Finland, and Sweden [36], Italy [36, 37], Netherlands [38], Uganda [39], India [40], Germany [41] and the Atlantic region [42, 43]. In European countries, a transition towards a healthier diet following the recommended guidelines and achieving nutritional adequacy has resulted to be the most sustainable option. Reductions in consumption of animal-based products are needed with differences according to country, sex, and food [32, 36–38, 41–43]. Major decreases in consumption of meat, snacks, and butter are needed in the Netherlands in conjunction with an increase in consumption of legumes, fish, nuts, and vegetables [38]. The Atlantic region diet has high GHG emissions, since it is based in livestock products and shellfish; however, it appears to have a high nutritional score mainly because of a low intake of sodium, added sugars and saturated fats [42, 43]. In Italy, changes towards a healthier diet in young population showed a reduction in CO_2_ emissions larger than 50% [37]. In Germany, 14-20% of the environmental burdens resulted from food losses along the value chain, out-of-home consumption was responsible for 8-28% impact, and animal products were shown to have caused the highest environmental burdens [41].

With respect to countries outside of Europe, findings differ. In the last few years, China has transitioned from staple-foods to non-staple foods and from plant-sourced foods to animal-sourced foods. Diets have suffered from globalization and become unhealthier and less sustainable, with meat and grains being the two dominant contributors to the carbon footprint. It has been proposed that returning to traditional dietary patterns would be a beneficial strategy to reduce environmental concerns, such as land use, GHG, etc. in China [33–35]. A similar situation has been observed in Uganda were urban residency and non-traditional dietary patterns have been negatively associated with environmentally sustainability compared to a more traditional (plant-based) dietary pattern [39]. On the contrary, in India, shifting to healthy guidelines has increased GHG emissions because the initial energy intake of the population was below recommendations, nonetheless, decreased environmental impacts were seen among those who currently meet dietary recommendations [40].

There are several studies focused on comparing the impact of changes in specific food products. Some studies have shown how diets with less animal products (beef, pork, poultry, and dairy products) and more plant-based products are beneficial for the environment [44–47] without compromising the health of the population and still meeting dietary recommendations [45, 46]. Women are more likely to consume ≤1 portion of meat a day compared with men and, also, females and older respondents (> 60 years) were more likely to hold positive attitudes towards animal welfare [44]. Studies that assessed specific foods founded that whole grain cereals, fruits, vegetables, legumes, nuts, and olive oil have been associated with improved health and have the lowest environmental impacts. Fish was associated with good health but was not simultaneously associated with less environmental impact, although it had a markedly lower impacts than red and processed meats which were associated with the largest increases in disease risk and environmental concerns [48]. Specifically, vegetables have been seen as one of the lowest impact food products, but it has been highlighted that the place where they are cultivated is important. For example, in the UK, importing seasonal vegetables from other countries in Europe has a lower impact than UK vegetables cultivated in heated greenhouses, despite the required transportation [49]. The environmental impacts of baby foods have also been assessed showing that meat-based ingredients cause almost 30% of the impacts [50].

Apart from the type of food used, the total amount of energy intake consumed must be considered when assessing sustainability, as it has done in this study when adjusting CO2 emissions per 1 kg of food products. Murakami et al. showed how considering energy intake, the inverse relation between the diet quality and de greenhouse gas emissions became stronger, specifically when measurements were done with the Mediterranean Diet score [51].

While the relationship between food consumption and sustainability is acknowledged, many are still not willing to change. It is for this reason that the consumer perception has been investigated by several studies [52–54]. Possible strategies to increase adherence to sustainable dietary practices and meet the United Nations agenda 2030 goal, such as supporting vegetarian dietary practices [55, 56], increasing the consumption of pulses [57], sustainable food systems in schools [58] and other strategies [59], have been put in practice. However, the United Nations agenda 2030 goals, specifically the target for GHG emissions, has not yet been reached. Future studies investigating optimal dietary patterns for both health and the environment, as well as strategies for how to increase awareness and consciousness to support population-based change are warranted to achieve the needed goals to be more sustainable and respectful with others and with the planet.

## Strengths and limitations

Recently, there has been an increase in research focusing on diet and sustainability. The current study contributes to the growing knowledge about an issue that is getting more importance every day. This is a strength of the present analyses, as it provides evidence to reinforce the health and sustainable impact of the Mediterranean diet. The large sample size used to calculate the dietary CO_2_ emissions is another strength. Moreover, once the CO_2_ calculations were done for each participant, an adjustment per kg of food product was done. This is a strength because it avoids the effect of the energy intake confounder. Calculating only the parameter of CO_2_ emissions for assessing the sustainability of a diet allows the impact to be observed independently from other parameters.

Limitations in relation to the present study also must be noted. This present analysis represents a cross-sectional study, and thus causal interferences cannot be established. Even though assessing CO_2_ alone is a strength, it can also be a limitation because of the lack of information representing the use of energy, land and water use, or other parameters which could be also used to assess sustainability. Finally, the population in this study was between 55 and 75 years old which might not make possible to extrapolate the results to a younger population.

## Conclusions

The current study shows that Mediterranean Diet can also be environmental protective since it appeared to be inversely related with GHG emissions, specifically CO_2_ emissions. In general, the higher the adherence to the Mediterranean Diet, the lower the total CO_2_ emissions showing that the erMedDiet index could be used pollution level index in the future. Findings may help inform and support public health initiative and dietary guidelines, such that recommendations continue to encourage making changes to food choices to achieve a healthier diet for both the population and the environment.

## Data Availability

Data described in the manuscript, code book, and analytic code will be made available upon request pending application and approval of the PREDIMED-Plus Steering Committee. There are restrictions on the availability of data for the PREDIMED-Plus trial, due to the signed consent agreements around data sharing, which only allow access to external researchers for studies following the project purposes. Requestors wishing to access the PREDIMED-Plus trial data used in this study can make a request to the PREDIMED-Plus trial Steering Committee chair: jordi.salas@urv.cat. The request will then be passed to members of the PREDIMED-Plus Steering Committee for deliberation.

## Abbreviations

BMI: Body mass index
CO_2_: Carbon Dioxide
EVOO: Extra Virgin Olive Oil
FFQ: Food frequency questionnaire
GHG: Greenhouse gas
GHGs: Greenhouse gases
MedDiet: The Mediterranean Diet
OR: Odds Ratio
SD: Standard deviations
Q1: Quartile 1
Q2: Quartile 2
Q3: Quartile 3
Q4: Quartile 4
17-item erMedDiet: 17-item energy-restricted Mediterranean dietary questionnaire
